# Study on the mechanism of KIF18B affecting the malignant progression of glioblastoma cells

**DOI:** 10.3389/fgene.2025.1540342

**Published:** 2025-03-05

**Authors:** Xiangyue Su, Liji Huang, Wei Ma, Rong Wang, Xiangjian Zeng, Gangliang Wei, Suli Mai, Min Yang, Shifu Tang

**Affiliations:** ^1^ Department of Laboratory Medicine, Key Laboratory of Precision Medicine for Viral Diseases, Guangxi Health Commission Key Laboratory of Clinical Biotechnology, Liuzhou People’s Hospital, Liuzhou, China; ^2^ Departments of Laboratory Diagnosis, Liuzhou Traditional Chinese Medical Hospital, Liuzhou, China

**Keywords:** KIF18B, bioinformatics, glioblastoma, prognosis, immune infiltration

## Abstract

**Background:**

Member of the driver protein family 18B (KIF18B) is a potential prognostic marker and is highly expressed in a variety of cancers. However, its function in glioblastoma (GBM) remains unclear.

**Methods:**

The expression data of KIF18B were obtained by accessing TCGA, CGGA and GEPIA databases, and verified by Western blot assay and immunohistochemistry. Glioma RNA sequencing data and clinical information were downloaded from TCGA and CGGA databases, and Kaplan-plotter survival analysis and Multivariable COX regression analysis were performed to plot ROC survival curves at 1, 3 and 5 years cBioPortal and MethSurv were used to carefully examine the prognostic value of KIF18B methylation. CBioPortal database and UALCAN database were used to obtain KIF18B co-expressed genes for GO and KEGG enrichment analysis, and gene set enrichment analysis (GSEA) software was used to explore the signaling pathway of KIF18B regulation of GBM. Finally, the correlation between KIF18B and GBM infiltration was studied by using TIMER database and TCGA dataset.

**Results:**

KIF18B was highly expressed in various cancers including GBM, and was positively correlated with glioma grade and negatively correlated with prognosis. Multivariable COX regression analysis and ROC curve showed that KIF18B was one of the independent risk factors for glioma prognosis. KIF18B methylation was negatively correlated with KIF18B expression, and the overall survival rate of patients with KIF18B hypomethylation was lower than that of patients with KIF18B hypermethylation. A total of 124 co-expressed genes were selected from the database. KEGG pathway analysis showed that KIF18B was mainly involved in the malignant progression of glioma through P53 and other signaling pathways. GSEA analysis showed that the high expression group of KIF18B was mainly enriched in E2F, G2M and other signaling pathways. The results of immunoassay showed that the expression of KIF18B was correlated with immune infiltration of tumor microenvironment.

**Conclusion:**

KIF18B is a key factor affecting the prognosis of GBM patients, and its targeting may provide a new therapeutic method for GBM patients.

## Introduction

Glioblastoma multiforme (GBM) represents the most common and aggressive variant of primary malignant brain tumors encountered in the adult population. It is notoriously characterized by an unfavorable prognosis (Yang et al., 2024; Thomas-Joulié et al., 2024). Despite the adoption of a multifaceted approach to therapeutic interventions, the overall survival (OS) rate for patients diagnosed with GBM has witnessed only modest advancements, with the median survival duration still failing to exceed 2 years (Thomas-Joulié et al., 2024). Among the elderly, this period is further abbreviated, with an average life expectancy of approximately 8 months following diagnosis (Villanueva-Meyer et al., 2017). Over the past decade, the rapid evolution of medical technologies has led to only incremental improvements in survival outcomes for individuals afflicted with GBM (Thomas-Joulié et al., 2024; Tupp et al., 2005; Wick et al., 2017). The challenges presented by GBM are diverse and complex, involving the intricate architecture of the blood-brain barrier (BBB), the immunosuppressive nature of the tumor microenvironment, and the pronounced heterogeneity of the neoplastic cells. These combined elements facilitate the swift and unrelenting progression of GBM, thereby undermining the efficacy of chemotherapeutic drugs, targeted treatment modalities, and immunotherapeutic strategies (Miller, 2022; Pombo Antunes et al., 2020; Liu et al., 2024). Despite the array of treatment options available for gliomas, substantial enhancements in clinical endpoints remain elusive. This is largely attributable to the difficulties associated with surgical resection, the aggressive tempo of disease advancement, and the high likelihood of tumor recurrence.

Kinesin family member 18B (KIF18B) is a member of the kinesin protein family and plays a crucial role in the regulation of mitotic processes and vesicle transport (Wu et al., 2018). The dysregulation of KIF18B has been associated with the initiation of uncontrolled cellular proliferation (Wu et al., 2018). At the commencement of mitosis, KIF18B localizes to the cellular cortex and interacts with microtubules and their adjacent areas, thereby regulating microtubule dynamics and promoting cytoskeletal reorganization during mitosis (Shrestha et al., 2023). In addition to its function in modulating microtubule length, KIF18B is implicated in the repair of DNA double-strand breaks (Luessing et al., 2021) and is responsible for the accurate positioning of the mitotic spindle (McHugh et al., 2018).

KIF18B has been shown to facilitate the growth and progression of multiple malignancies. For example, in renal clear cell carcinoma, KIF18B is markedly overexpressed and correlates with a less favorable prognosis and advanced tumor stage (Liu Q. et al., 2020). Additionally, a distinct study confirmed that the reduction in KIF18B levels can alter the Akt/GSK-3β/β-catenin signaling pathway, Thus inhibiting the proliferation and invasion of breast cancer cells and increasing their sensitivity to chemotherapy drugs (Jiang et al., 2021). Recently, Liu et al. reported that the inhibition of KIF18B or the administration of T09, a radiosensitizer targeting KIF18B, significantly enhances the radiosensitivity of sarcoma cells, delays tumor growth in both subcutaneous and orthotopic xenograft models, and prolongs the survival of murine subjects (Liu W. et al., 2020). In colorectal cancer, KIF18B has been observed to interact with SP1, resulting in the upregulation of PARPBP and the maintenance of oxaliplatin resistance in oxaliplatin-resistant colorectal cancer (OR-CRC) cells (Hong et al., 2021). Nevertheless, the expression profile, prognostic relevance, and biological functions of KIF18B in glioblastoma multiforme (GBM) have not been comprehensively investigated. Hence, the present study was designed to examine the expression of KIF18B in GBM and its clinical implications using mRNA expression data from the TCGA (Wang et al., 2016) and CGGA (Zhao et al., 2021) databases, with the goal of identifying novel therapeutic targets and advancing glioma research.

## Materials and methods

### Data collection

The human glioma cell lines A172, U87, and U251 were purchased from the National Certified Cell Culture Preservation Center and cultured in DMEM (Hyclone) supplemented with 10% fetal bovine serum. The normal astrocyte line SVG p12 was purchased from the United States Typical Culture Preservation Center and cultured in EMEM (ATCC) supplemented with 10% fetal bovine serum. The central nervous system (CNS) tissue microarray was purchased from Zhongke Guanghua Intelligent. The microarray assembly comprises total of 109 individual samples, which include a diverse array of pathological conditions: seven samples of WHO grade I astrocytoma, 32 samples of WHO grade II astrocytoma, 23 samples of WHO grade III anaplastic astrocytoma, 36 samples glioblastoma, and 11 samples of normal CNS tissue.

### The main reagent

The KIF18B protein was purchased from Abcam Company. Trypsin, non-programmed cell cryopreservation solution, SDS-PAGE gel rapid configuration kit, and hematoxylin dye solution were purchased from Beijing Lanjike Technology Co., Ltd. The whole protein extraction kit, mouse anti-GAPDH monoclonal antibody, goat anti-mouse IgG/horseradish peroxidase conjugate, goat anti-rabbit IgG/horseradish peroxidase conjugate, and DAB color developing solution were purchased from Beijing Zhongshan Jinqiao Biotechnology Co., Ltd. SDS-PAGE loading buffer (5X), ECL chemiluminescent solution, penicillin-streptomycin mixture (100X), and fetal bovine serum were purchased from Biyantian Biotechnology Co., Ltd. EMEM high-glucose medium was purchased from the American Type Culture Collection, and DMEM high-glucose medium was purchased from Thermo Fisher Scientific.

### Data source and processing

The expression of the KIF18B gene in different tumor types was detected in the TIMER (Li et al., 2020) database and the UCSC database (https://xenabrowser.net/). The expression of KIF18B in gliomas was analyzed on the website for Interactive GEPIA (Tang et al., 2017). After downloading glioma tissue sequencing data and clinical information from the TCGA database and the CGGA database, a gene expression matrix was generated. Correlation analysis was performed after excluding cases with unknown or incomplete clinicopathological characteristics and those lacking prognostic follow-up data. The correlation between the KIF18B expression level and glioma grade was analyzed, respectively. Kaplan-Meier survival analysis, clinical correlation analysis, and multivariable Cox regression analysis were further conducted. The receiver operating characteristic (ROC) survival curves for 1, 3, and 5 years were calculated.

### Western blotting

Protein was extracted using RIPA lysis buffer, and the protein concentration was determined by BCA protein assay according to the manufacturer’s instructions. The protein was then aliquoted, sealed, and stored at −80°C for later use. After adding protein loading buffer, the samples were denatured in a metal bath at 100°C for 10 min. The gel was prepared according to the instructions provided. Following gel electrophoresis, the proteins were transferred onto a nitrocellulose membrane. The transferred nitrocellulose membrane was placed in a sealed container and washed with TBST for 3 min before blocking with 5% skim milk in a room temperature shaker for 60 min. After blocking, the membrane was washed again with TBST for 5 min. The primary antibody working solution was added, and the membrane was incubated overnight at 4°C. The TBST wash solution was used to clear the membrane three times for 5 min each. The secondary antibody, conjugated to horseradish peroxidase, was incubated at room temperature for 2 h. After washing with TBST, the imprinted bands were visualized using ECL reagent. The image was acquired using an iBright 1,500 Imaging System (Thermo Fisher Scientific) and the band intensities were quantified using ImageJ software.

### Immunohistochemistry

Proteins were extracted using RIPA lysis buffer, and protein concentrations were determined by BCA protein assay according to the manufacturer’s instructions. The proteins were then aliquoted, sealed, and stored at −80°C for subsequent use. After adding protein loading buffer, the samples were denatured in a metal bath at 100°C for 10 min. The SDS-PAGE gel was prepared according to the provided instructions. Following electrophoresis, the proteins were transferred onto a nitrocellulose membrane. The transferred membrane was placed in a sealed container and washed with TBST for 3 min before blocking with 5% non-fat milk in a room temperature shaker for 60 min. After blocking, the membrane was washed again with TBST for 5 min. The primary antibody working solution was added, and the membrane was incubated overnight at 4°C. The membrane was then washed three times with TBST for 5 min each. The HRP-conjugated secondary antibody was incubated at room temperature for 2 h. After washing with TBST, the immunoreactive bands were visualized using an ECL detection reagent. The image was acquired using an iBright 1,500 Imaging System (Thermo Fisher Scientific), and band intensities were quantified with ImageJ software.

### DNA methylation and genetic change analysis

In the cBioPortal (https://www.cbioportal.org/), we explored the frequency of KIF18B alterations across various cancer types from the TCGA dataset, including mutation types, CNA (copy number alterations), and KIF18B methylation data. The data of KIF18B homologous gene KIF18A were obtained from CGGA database to study the correlation between its expression and methylation. The SMART online tool (SMART, http://bioinfo-zs.com/) was used to validate the correlation between KIF18B methylation levels and KIF18B mRNA expression. Additionally, the MethSurv online tool (https://biit.cs.ut.ee/methsurv/) was utilized to investigate the prognostic value of KIF18B methylation levels in glioma patients.

### Analysis of co-expression genes of KIF18B in GBM and its related mechanisms

The cBioPortal database, CPTAC Database (https://cptac-data-portal.georgetown.edu/datasets) and the UALCAN database (Chandrashekar et al., 2017) were used to obtain KIF18B co-expressed genes. The STRING database (von Mering et al., 2003) was utilized to analyze the protein-protein interaction (PPI) networks of these co-expressed genes. Furthermore, gene ontology (GO) function enrichment and Kyoto Encyclopedia of Genes and Genomes (KEGG) pathway analysis for the co-expressed genes were conducted using the DAVID database (Dennis et al., 2003).

### Gene set enrichment analysis (GSEA)

GSEA (Subramanian et al., 2005) is a method used to analyze whole-genome expression profile data from microarrays. By examining gene expression profiles, we can determine the extent to which specific functional gene sets are expressed and whether this expression is statistically significant. Using the gene expression matrix from the TCGA data, we divided the samples into high and low expression groups based on the median expression level of KIF18B. GSEA was then performed to identify enriched signaling pathways in the high expression group of KIF18B. For each analysis, the genomes were permuted 1,000 times. Enriched pathways were identified based on the false discovery rate (FDR) and normalized enrichment score (NES).

### Immune infiltration analysis

Based on the bioinformatics analysis platform Sangerbox 3.0 (http://sangerbox.com/), data screening was performed: (1) immune regulatory gene analysis (2) Input gene: KIF18B, (3) Data source: TCGA + GTEx, (4) Sample source: For all samples, (5) data transform: log2 (x + 0.001). Immune checkpoint analysis, Various algorithms in immune cell analysis such as: ESTIMATE algorithm, CIBERSORT algorithm, xCELL algorithm as above. We then calculated the correlation between high and low KIF18B expression groups and immune checkpoint genes and immune cells. The TIMER database is dedicated to molecular characterization of tumor-immune interactions and predicts infiltration of immune cell subpopulations in more than 10,000 tumor samples from 32 types of cancer. We conducted an online analysis of the correlation between KIF18B expression level and Immune cell infiltration in GBM tumor microenvironment through the Immune Association module of TIMER database, and further analyzed the role of KIF18B in GBM tumor-related immune cell infiltration.

### Statistical analysis

Correlations between variables were explored using Pearson or Spearman coefficients. Continuous variables fitting a normal distribution between binary groups were compared using a t-test. Otherwise, the Mann-Whitney U test was applied. Categorical variables were compared using the chi-squared test. Survival curves for prognostic analyses of categorical variables were generated using the Kaplan-Meier method, while the log-rank test was applied to estimate statistical significance. Multivariable Cox regression analysis was used to assess whether KIF18B expression and clinical features (age, sex, tumor grade, chemoradiation therapy, IDH mutation status, methylation status, and 1p/19q codeletion) were risk factors for the prognosis of glioma patients. The criteria for determining significant enrichment in GSEA analysis were NES >1.5, FDR q-val <0.01, and NOM p < 0.05. GraphPad Prism 6.0 software and SPSS 26.0 software were used for plotting and statistical analysis, respectively. All the above statistical methods have statistical significance when p < 0.05, and this value does not need to be adjusted for any comparison.

## Results

### KIF18B is highly expressed in GBM

**FIGURE 1:**
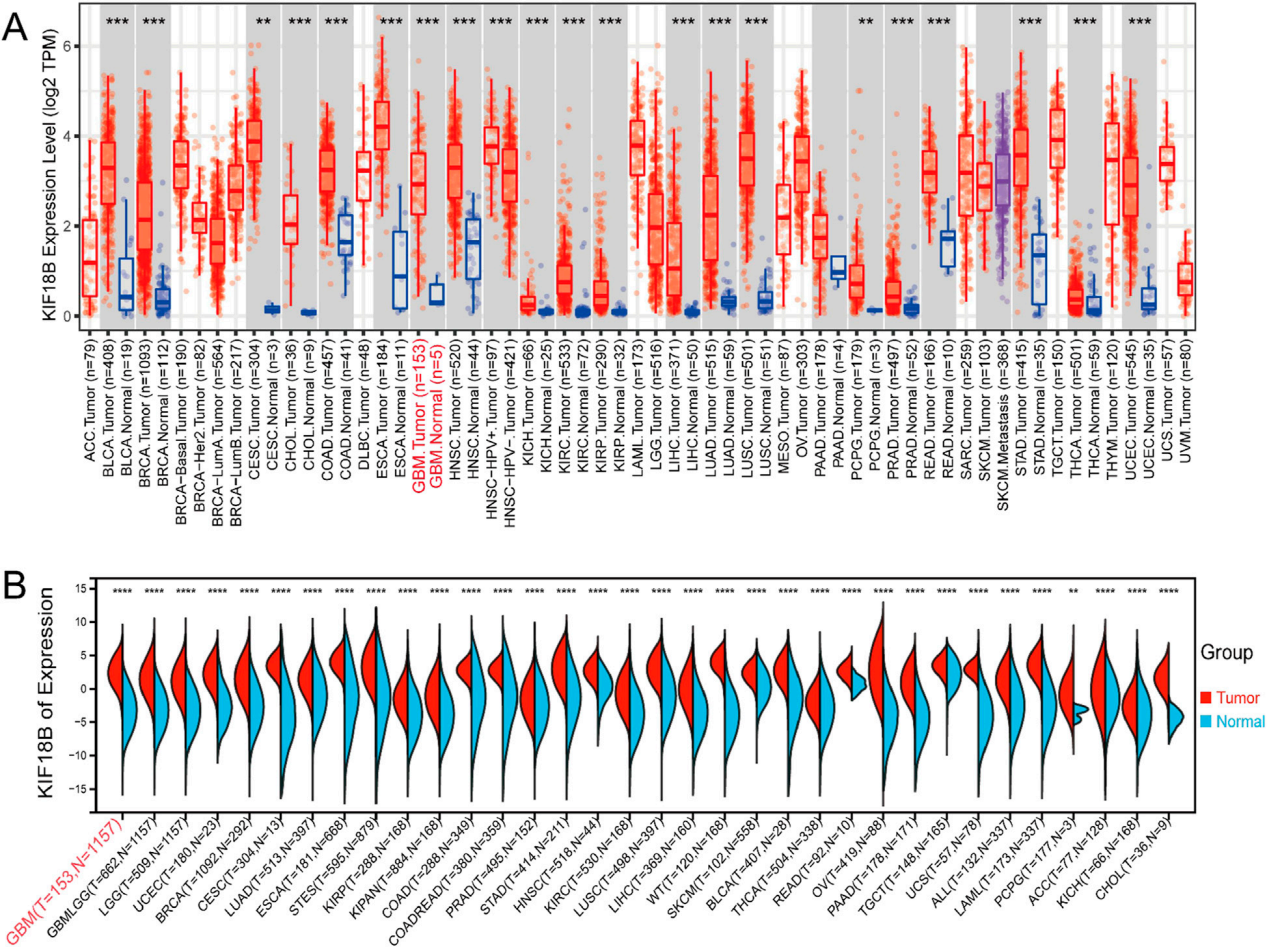
High expression of KIF18B in Glioma **(A)** expression of KIF18B in 32 cancer tissues and their adjacent tissues in the TIMER database, **(B)** expression of KIF18B in 34 cancer tissues and normal tissues in the UCSC database; The red font appears as GBM. *p < 0.05, **p < 0.01, ***p < 0.001, ****p < 0.0001.

**FIGURE 2:**
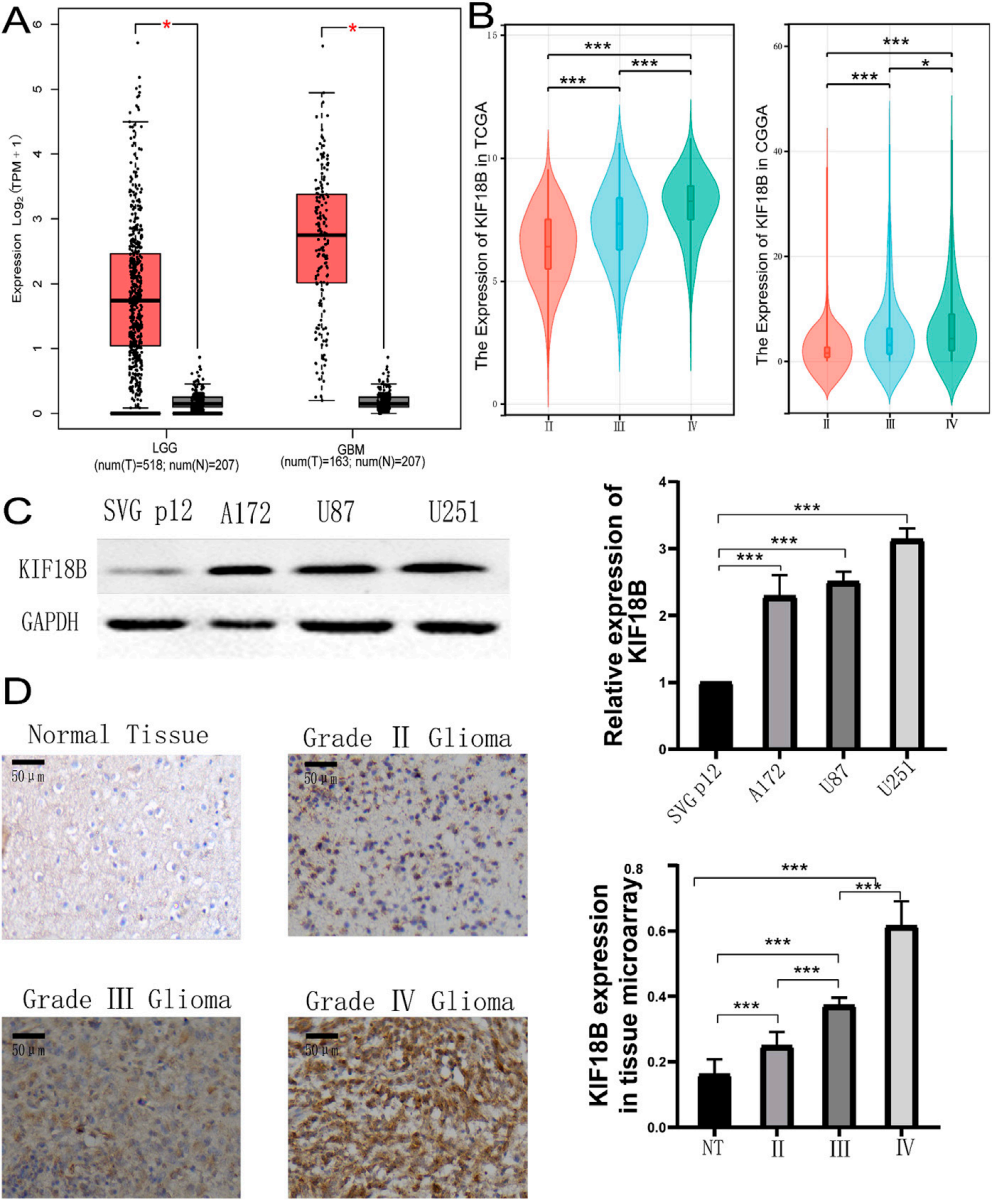
Expression of KIF18B in Gliomas **(A)** KIF18B expression levels between gliomas and normal tissues in the GEPIA dataset, **(B)** KIF18B mRNA expression and glioma grade, **(C)** The expression level of KIF18B in GBM cells U251, U87, A172 and normal glial cells SVG p12, **(D)** Expression of KIF18B in glioma of different grades. *p < 0.05, **p < 0.01, ***p < 0.001.

Online analysis of KIF18B transcription levels in tumor and normal tissues using the TIMER database showed that KIF18B was highly expressed in a variety of fatal cancers, including GBM (P < 0.05) (Figure 1A). In addition, to further confirm the expression of KIF18B in different cancers, we analyzed its expression in tumor tissues using the UCSC online platform. The results indicated that KIF18B was highly expressed in various tumors, including GBM (Figure 1B). To more directly analyze the expression level of KIF18B in gliomas, we accessed the GEPIA database. The results revealed that KIF18B expression in gliomas was significantly increased, with higher expression in GBM compared to LGG (p < 0.05, Figure 2A). Additionally, the CGGA and TCGA datasets showed that higher expression of KIF18B correlated with higher glioma grade (p < 0.05, Figure 2B). We also verified the expression of KIF18B in GBM cells by Western blot, and the results confirmed that the expression of KIF18B in GBM cells was higher than that in normal glial cells (p < 0.05, Figure 2C). In addition, it was also confirmed by tissue chip that the expression of KIF18B was positively correlated with the grade of glioma (p < 0.05, Figure 2D; Supplementary Figure S1), which could be mutually verified with the results of bioinformatics data analysisThese findings suggest that KIF18B expression is involved in the malignant development of glioma. Therefore, we speculate that high expression of KIF18B may also be associated with poor prognosis in glioma patients.

### High expression of KIF18B predicted poor prognosis in GBM patients

**FIGURE 3:**
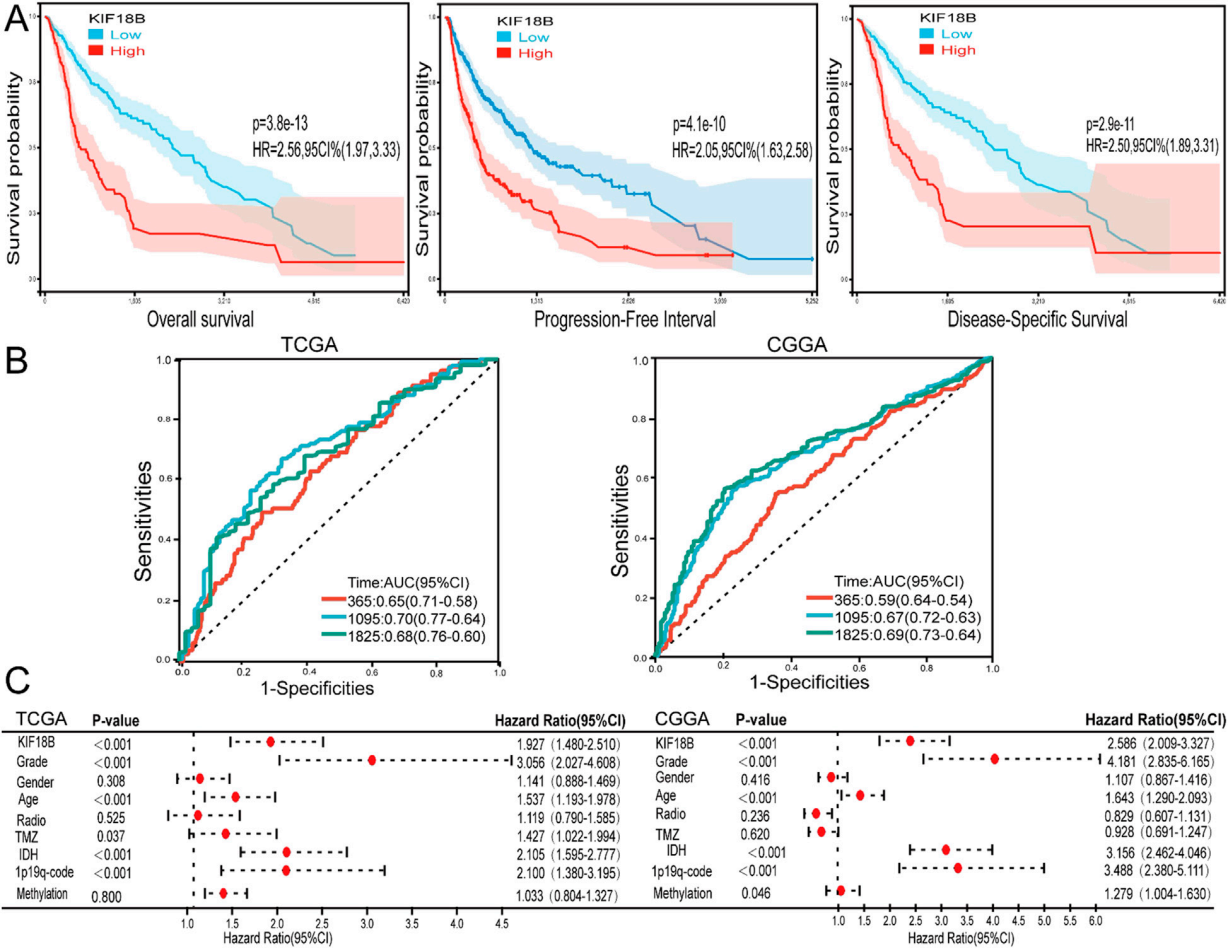
High expression of KIF18B predicts poor prognosis in GBM patients **(A)** The effects of KIF18B expression in TCGA database on OS, PFI and DSS in glioma patients, **(B)** 1, 3 and 5-year ROC curves of glioma patients in CGGA and TCGA databases, **(C)** Forest maps of KIF18B expression and patients’ clinical characteristics were analyzed by multivariable Cox based on TCGA and CGGA databases.

To investigate whether high expression of KIF18B is associated with poor prognosis in glioma patients, we obtained prognostic follow-up data from the TCGA dataset. The results indicated that in glioma patients, overall survival, progression-free interval, and disease-specific survival were significantly lower in patients with high KIF18B expression compared to those with low expression (Figure 3A). ROC curve analysis revealed that KIF18B could serve as a marker to predict 1-year, 3-year, and 5-year overall survival, with AUC values of 0.65, 0.73, and 0.70 based on the TCGA dataset, and 0.59, 0.67, and 0.69 based on the CGGA dataset, respectively (Figure 3B). Multivariable Cox regression analysis of the CGGA dataset identified KIF18B expression level, grade, age, IDH mutation status, 1p19q co-deletion, and methylation status as independent prognostic factors. Similarly, multivariable Cox regression analysis of the TCGA dataset revealed that KIF18B expression level, grade, age, chemotherapy status, IDH mutation status, and 1p19q co-deletion were independent prognostic indicators (Figure 3C).

### Hypomethylation is associated with KIF18B expression and poor prognosis in gliomas

**FIGURE 4:**
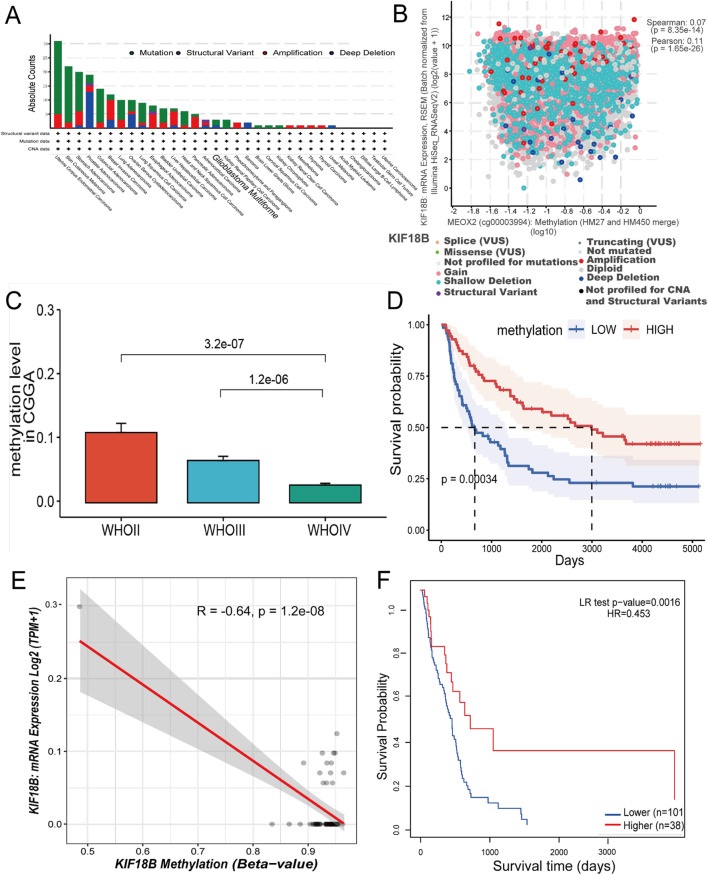
Analysis of KIF18B gene copy number variation and DNA methylation **(A)** Variation frequency and mutation type of KIF18B in cancer, **(B)** The correlation between KIF18B expression and its methylation was found in the cBioPortal database, **(C)** Correlation between glioma grade and KIF18A methylation level in CGGA data **(D)** Overall survival curve based on KIF18A methylation level grouping, **(E)** Correlation between KIF18B methylation level and expression **(F)** Overall survival curve based on KIF18B methylation level grouping.

Upon verification of KIF18B expression, subsequent efforts were directed towards elucidating the underlying mechanisms of its elevated expression. Initially, the cBioPortal database was employed to ascertain KIF18B genetic alterations across various tumor samples within the TCGA dataset. It was observed that mutations in KIF18B were predominant in cancer cases, notably in GBM (Figure 4A). Subsequent analysis of the clinical implications of these genetic modifications in gliomas revealed a partial correlation between KIF18B expression and its methylation status in mutated instances (Figure 4B). To further substantiate these findings, the correlation between the methylation of KIF18A, a homologous gene to KIF18B, and glioma grade was investigated using the CGGA database. The analysis indicated an inverse relationship between the methylation level of KIF18A and glioma grade (Figure 4C). Survival analysis demonstrated that patients exhibiting higher methylation levels tended to have a more favorable OS (Figure 4D). In addition, to describe this association more precisely, we examined methylation levels at the cg24545250 probe site. Figure 4E Results showed that the methylation level of KIF18B was negatively correlated with its mRNA expression. Similarly, glioma patients characterized by KIF18B hypomethylation had a poor prognosis, as shown in Figure 4F.

These results suggest that KIF18B hypomethylation may be associated with high KIF18B expression and poor prognosis in glioma patients.

### Co-expression gene and functional enrichment analysis of KIF18B

**FIGURE 5:**
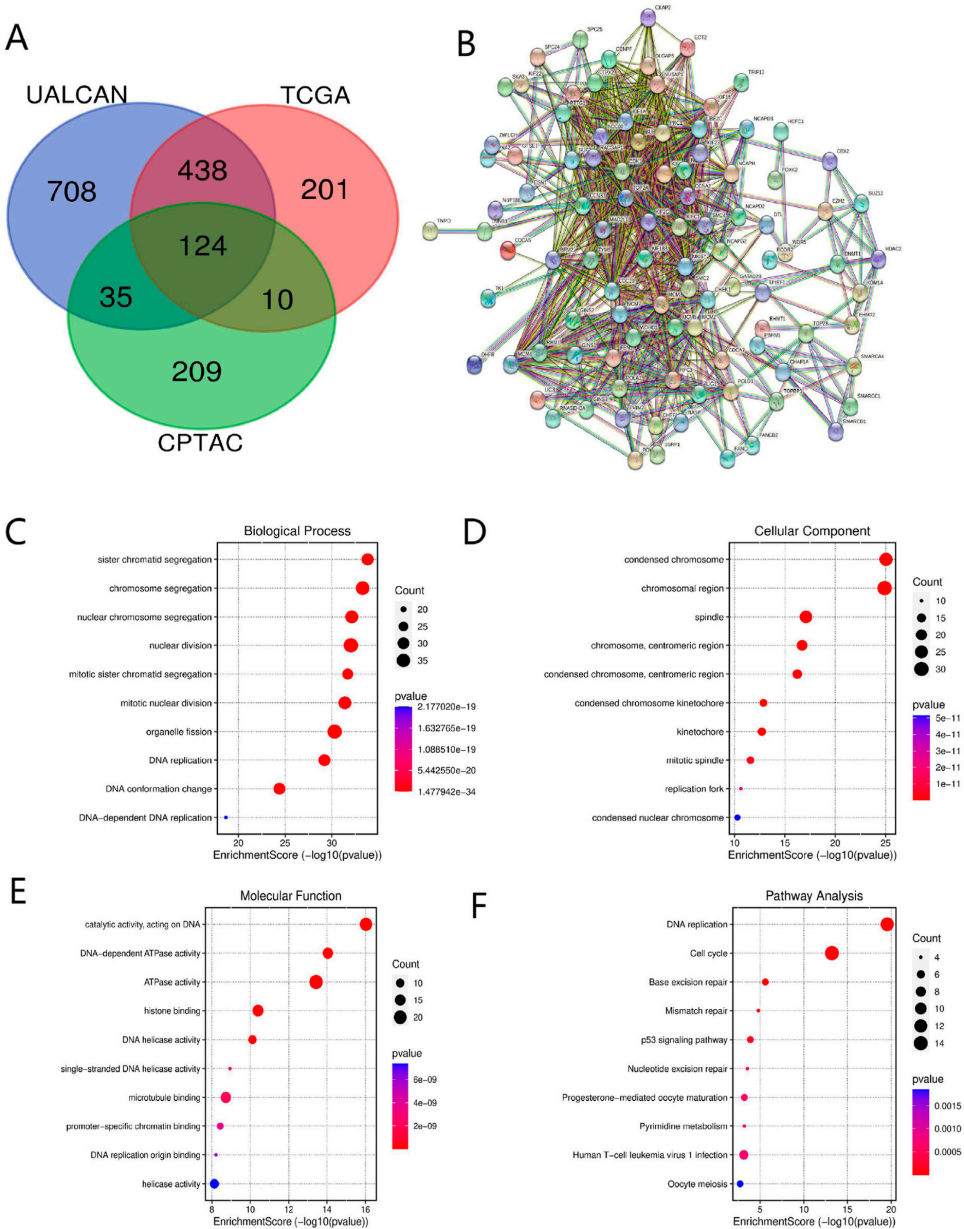
Co-expressed genes and functional enrichment analysis of KIF18B **(A)** Co-expressed genes, **(B)** PPI, **(C–E)** GO analysis, **(F)** KEGG pathway analysis.

Based on the UALCAN database, we identified 1,305 KIF18B-related genes in GBM (Pearson’s correlation coefficient >0.5, P < 0.05). A total of 773 and 378 KIF18B-related genes were obtained from the TCGA and CPTAC databases, respectively, using the cBioPortal platform (Pearson’s correlation coefficient >0.5, P < 0.05). We then screened for 124 co-expressed genes common to all three databases (Figure 5A). The STRING tool was employed to predict protein-protein interactions among the co-expressed genes. With a minimum correlation of 0.4, the PPI network comprised 123 nodes and 2,661 edges (P < 0.001) (Figure 5B). Functional enrichment analysis of the 124 differentially expressed genes was conducted using DAVID. The results indicated that the main biological processes (BP) associated with the co-expressed genes included sister chromatid separation, mitotic nuclear division, DNA replication, etc*.* (Figure 5C). Cellular component (CC) localization was primarily in the chromosome, centromere, and spindle apparatus (Figure 5D). The main molecular functions (MF) involved catalytic ATPase activity, histone binding, and microtubule binding (Figure 5E). KEGG pathway enrichment analysis revealed that the co-expressed genes were predominantly involved in pathways such as DNA repair, cell cycle, and P53 signaling (Figure 5F).

### Gene set enrichment Analysis (GSEA)

**FIGURE 6:**
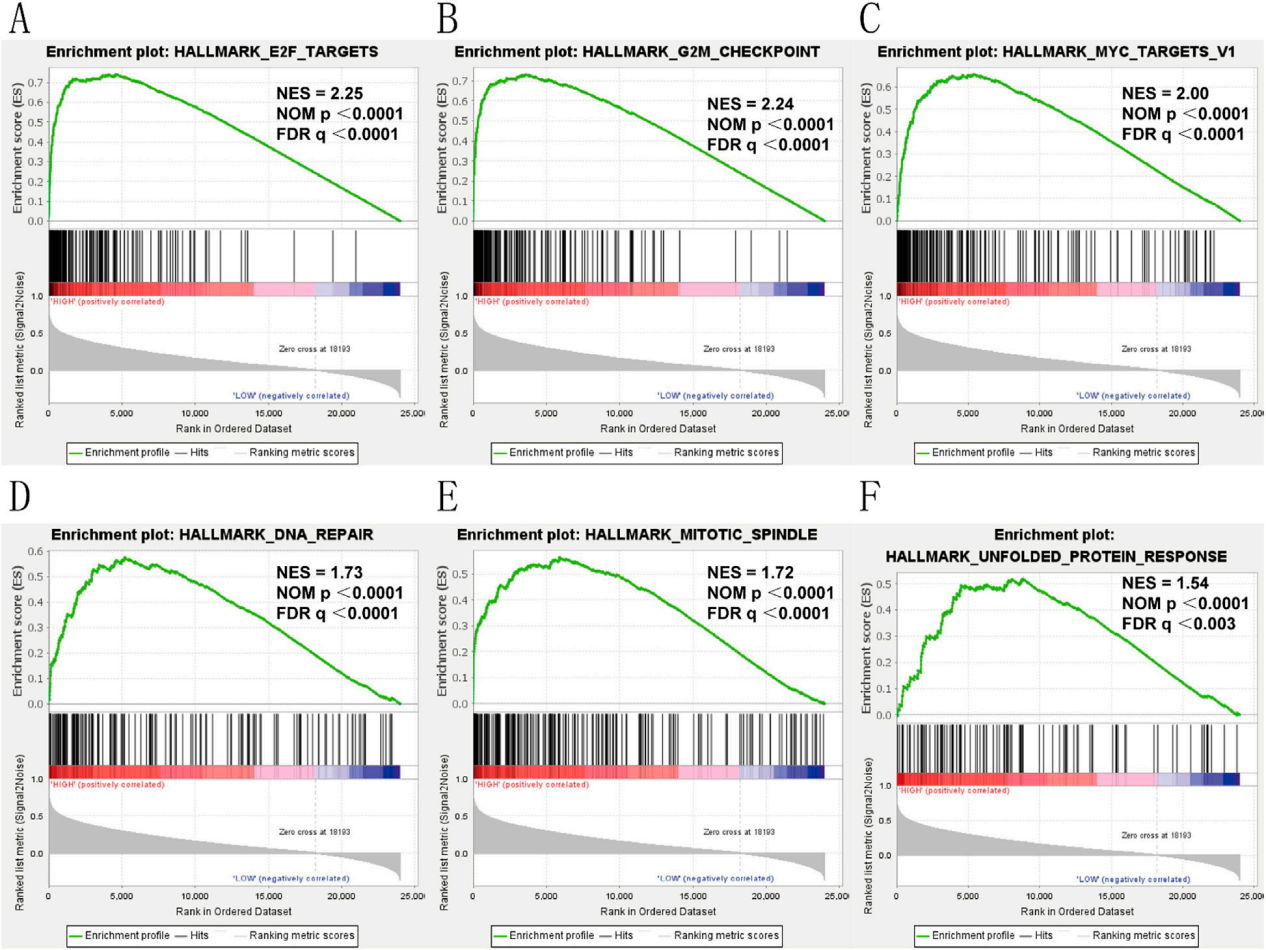
Gsea **(A)** E2F-Trrgets, **(B)** GP2M-checkpoint, **(C)** MYC-Trrgets, **(D)** DNA-Rrpair, **(E)** mitotic spindle, **(F)** unfolded protein response.

To elucidate the role of KIF18B in glioma, we divided glioma samples into high and low expression groups based on the median expression value of KIF18B in the TCGA dataset. We then performed GSEA to identify significantly activated pathways in glioma patients with high KIF18B expression compared to those with low expression. The results revealed that in GBM patients with high KIF18B expression, the following signaling pathways were significantly activated: E2F Targets (Figure 6A), G2M Checkpoint (Figure 6B), MYC Targets (Figure 6C), DNA Repair (Figure 6D), Mitotic Spindle (Figure 6E), and Unfolded Protein Response (Figure 6F) (NES >1.5, FDR q-val <0.01, and NOM p < 0.01).

### Relationship between KIF18B and immune infiltration of GBM

**FIGURE 7:**
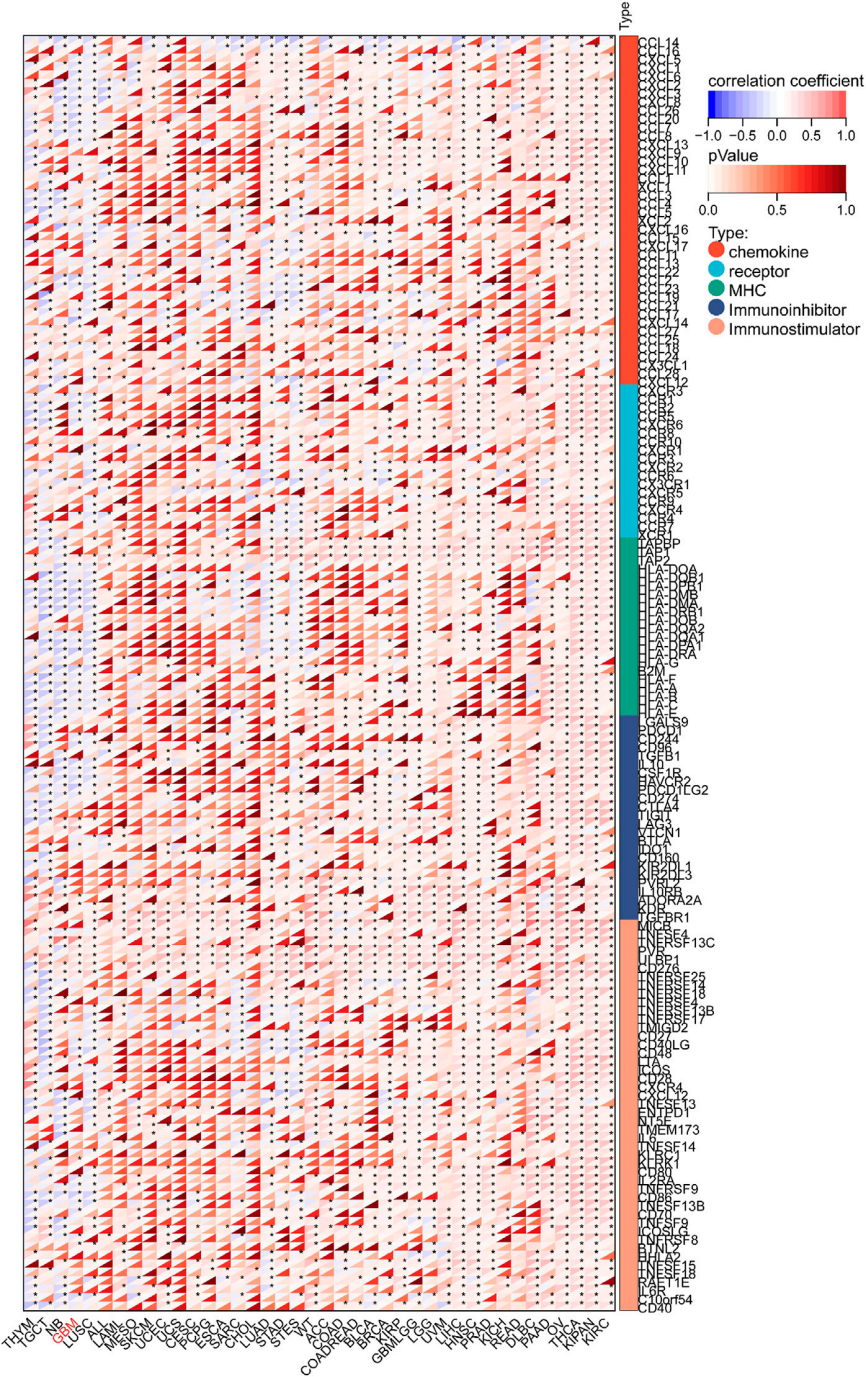
Correlation between KIF18B and 122 immunomodulators (chemokines, receptors, MHC, and immunostimulators.*p < 0.05; Red represents positive correlation, blue represents negative correlation.

**FIGURE 8:**
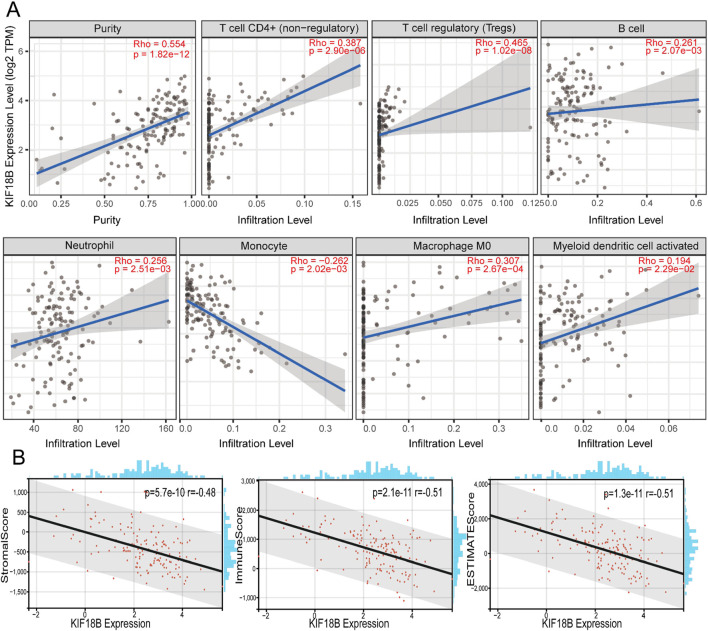
Correlation between the KIF18B expression level and infiltration of the tumor microenvironment **(A)** Correlation analysis of KIF18B mRNA expression with immune infiltration based on TIMER databases, **(B)** Correlation between KIF18B mRNA expression and stomalscore, Immunescore, ESTIMTEscore.

We targeted the KIF18B gene in the GTEx dataset from TCGA and extracted 150 immunostimulator [including chemokine (41), receptor (18), MHC (21), immunoinhibitor (24), and immunostimulator (46)] and 60 immune checkpoints (Inhibitory (24) and Stimulatory (36) marker genes). The results showed that KIF18B was highly correlated with most immune checkpoints genes and immune checkpoints in GBM (Figure 7; Supplementary Figure S2). Beyond that, the expression of immune checkpoint molecules in glioma patients with high expression of KIF18B was higher than that in glioma patients with low expression of KIF18B (Supplementary Figure S3). In addition, we also used the xCELL algorithm to estimate the infiltration level of tumor-infiltrating immune cells (TICs) in the tumor microenvironment (TME). The results showed that KIF18B was negatively correlated with most TICs in GBM, but highly positively correlated with TH2 cells (Supplementary Figure S4). To further elucidate the tumor immune function of KIF18B, we used the TIMER2.0 database to analyze the correlation between KIF18B mRNA expression and GBM immune cell infiltration levels. The results showed that the expression of KIF18B was positively correlated with CD4+ T cells (rho = 0.387, p = 2.90e-06), Tregs (rho = 0.465, p = 1.02e-08), B cells (rho = 0.261, p = 2.07e-03), neutrophils (rho = 0.256, p = 2.51e-03), macrophages (rho = 0.307, p = 2.67e-04), and dendritic cells (rho = 0.194, p = 2.29e-02). It was negatively correlated with dendritic cells (rho = −0.262, p = 2.02e-03) (Figure 8A). Similarly, the use of the CIBERSORT algorithm can lead to similar conclusions, indicating a significant difference in the expression of KIF8B between immune cells in the tumor microenvironment (Supplementary Figure S5). We also used the ESTIMATE algorithm score to predict tumor purity, and the results showed that KIF18B expression was negatively correlated with stromal score, immune score, and ESTIMATE score (Figure 8B).

In summary, the overexpression pattern of KIF18B is TME-specific, suggesting the potential of KIF18B as a target for standardized cancer immunotherapy. The immunosuppressive effect of KIF18B on the TME was most pronounced in GBM, suggesting that KIF18B may be a suitable candidate for anti-KIF18B immunotherapy.

## Discussion

Despite progress in medical treatments, the curative rate for patients with GBM has not achieved satisfactory levels, with challenges such as tumor metastasis, invasion, and drug resistance posing substantial hurdles. Consequently, the identification of prognostic markers and key therapeutic targets for GBM is critically important. Emerging research has increasingly highlighted the association between KIF18B and various cancers, with accumulating evidence suggesting that KIF18B is overexpressed in multiple malignancies affecting the respiratory, urinary, and digestive systems, and that it facilitates tumor proliferation. In the current investigation, a comparative analysis of tumor and normal tissue data across several databases revealed a significant correlation between the upregulation of KIF18B and diverse cancers, including GBM. The expression level of KIF18B in GBM was observed to be significantly higher compared to that in LGG and normal brain tissue. This observation was corroborated by Western blot analysis, which demonstrated that the expression of KIF18B in GBM cells was considerably increased relative to normal glial cells. To further validate these findings at the tissue level, immunohistochemical staining was utilized to evaluate KIF18B expression in GBM tissues, revealing elevated expression compared to normal brain tissues and a positive correlation with glioma grade.

Oncogenes are often linked to the prognosis and clinical features of cancer patients. Therefore, the objective of this study was to elucidate the relationship between KIF18B expression and the clinical and molecular characteristics of glioma. Kaplan-Meier survival analysis, ROC curves for 1, 3, and 5-year survival intervals, and multivariate Cox regression analysis all consistently indicated that KIF18B may function as an independent predictor of unfavorable prognosis in glioma patients.

This investigation marks the first utilization of the cBioPortal database to analyze the genetic alterations of KIF18B in glioma, with mutation being the predominant type. It is widely acknowledged that gene mutation constitutes a pivotal factor in the onset of cancer. Moreover, the genetic information encoded within DNA sequences encompasses a level of genetic complexity, and the study of epigenetic modifications—including non-coding RNAs, DNA methylation, histone modifications such as methylation, acetylation, phosphorylation, ubiquitination, and glycosylation—has elucidated novel mechanisms governing genetic information regulation. These findings afford a novel perspective for a more nuanced comprehension of the intricate network of genetic regulation (Mattei et al., 2022). A notable molecule in glioma is MGMT, whose promoter methylation status serves as an independent prognostic indicator. Specifically, patients exhibiting MGMT promoter methylation exhibit a prolonged median survival time compared to those without such methylation (Mansouri et al., 2019; Della Monica et al., 2022). The bioinformatics outcomes of the present study suggest that the hypomethylation of the KIF18B gene may be implicated in the upregulated expression of KIF18B in gliomas, and individuals with hypomethylated KIF18B gene status experience a less favorable prognosis. However, the empirical verification of this hypothesis extends beyond the scope of the current investigation. Should the opportunity arise, it would be beneficial to conduct further experimental studies to validate this conclusion.

In prostate, breast, and lung cancers, the proliferative regulation of tumor cells by KIF18B is contingent upon the activation of the AKT pathway (Wu et al., 2021; Chen et al., 2024). In contrast, in hepatocellular carcinoma, cervical cancer, and bladder urothelial carcinoma, the modulation of tumor cell viability by KIF18B is associated with the Wnt/β-catenin pathway (Wu et al., 2018; Zhang and Liu, 2023; Liu et al., 2022). This indicates that the oncogenic function of KIF18B in cancer cells is mediated through the collaborative engagement of diverse signaling pathways. Elucidating the direct downstream targets and mechanisms of KIF18B is essential for a comprehensive understanding of tumor progression dynamics.

To delineate the biological role of KIF18B in glioma, we performed GO, KEGG analysis, and GSEA. The results indicated that KIF18B primarily regulates the malignant progression of glioma through cellular functions related to mitosis, cell cycle regulation, and the Rb-E2F signaling pathway. Under physiological conditions, the equilibrium between cell self-renewal and apoptosis is maintained, thereby preserving homeostasis and preventing uncontrolled cell growth and proliferation. However, upon exposure to carcinogenic stimuli, disruption of the cell cycle can result in abnormal cell proliferation and cancer development (Yousefi et al., 2024; Zheng et al., 2024). The E2F transcription factor is a critical component in the Rb-E2F pathway, governing cell cycle progression through the action of cyclin-dependent kinases (CDKs). In quiescent cells, the Rb is maintained in a hypophosphorylated state, sequestering E2F and repressing the transcription of genes crucial for mitosis, thus impeding progression through the G1/S cell cycle checkpoint. In activated cells, growth factor stimulation leads to the activation of Cyclin D1 and CDKs, resulting in the phosphorylation of Rb during late G1 phase, the release of E2F, and the subsequent transcriptional activation of genes required for DNA synthesis and cell growth, thereby promoting GBM cell proliferation and growth (Almalki, 2023). GSEA revealed that the upregulation of KIF18B can enhance the activation of the cell cycle and Rb-E2F signaling pathway, which is consistent with the tumor-related regulatory mechanisms documented in the literature. Based on these findings, it is justified to conclude that KIF18B influences the malignant progression of glioma via these cancer-relevant signaling pathways. But unfortunately, this paper can not be verified by cell cells.

Tumor cells are capable of expressing, secreting, or inducing a array of immunosuppressive molecules that impede the body’s anti-tumor immune response. The interaction between immune checkpoint molecules and their corresponding ligands can inhibit the activation and efficacy of T cells, thereby facilitating the evasion of tumor immunity. In the face of proliferating tumor cells, the body emits “danger signals” to recruit lymphocytes aimed at tumor elimination. Concurrently, tumor mutations can prompt the establishment of an immunosuppressive tumor microenvironment (Bader et al., 2020), which includes the enlistment of immunosuppressive cells such as myeloid-derived suppressor cells and regulatory T cells. Research has demonstrated that regulatory T cells can suppress the anti-cancer functions of CD8+ T cells and CD4+ T cells, thereby modulating the anti-tumor response (Chen et al., 2020). Moreover, M2 macrophages contribute to the inhibition of tumor immune defense mechanisms (Cheng et al., 2019). Consequently, it is imperative to ascertain the impact of KIF18B on the GBM tumor immune microenvironment. Our findings indicate that KIF18B is inversely associated with the majority of immunomodulatory genes and immune checkpoints in GBM. This suggests that KIF18B may suppress tumor immune responses by facilitating the infiltration of immune cells. Further analysis revealed a high correlation between KIF18B and most TIs) in GBM. Additionally, the ESTIMATE immune algorithm confirmed that higher expression levels of KIF18B correspond to lower immune, tumor-infiltrating lymphocyte, and ESTIMATE scores. Therefore, it is promising that the inhibition of KIF18B could modulate the immune microenvironment, thereby providing a therapeutic target for GBM.

## Conclusion

Through an integrative approach that combines classic experimental methodologies with sophisticated bioinformatics analyses, we have ascertained that KIF18B is upregulated in GBM and its expression level increases concomitantly with the advancement of tumor grade. Furthermore, heightened expression of KIF18B is associated with adverse patient prognoses and emerges as an independent prognostic risk factor for patients with GBM. Our results demonstrate for the first time the key role of KIF18B in the process of glioma canceration, thus proving that it is highly likely to be used as a potential therapeutic target for the treatment of glioma, igniting new hopes for improving the survival and prognosis of patients with glioma. Unfortunately, the investigation into the upstream regulatory elements and the *in vivo* mechanisms that control KIF18B expression is still in its infancy. Unraveling the molecules or extrinsic factors capable of inducing the transcriptional activation of KIF18B represents a critical frontier that requires further scientific inquiry. More regrettably, most of the conclusions in this paper are inferred from multiple databases, and there is no further experiment to prove it. However, this paper also provides a new idea for the discovery of tumor markers of GBM, which is worthy of further study. We hope that our current findings will serve as a catalyst for future research and that the insights gained from our bioinformatics analysis will be validated and expanded upon through subsequent experimental studies. We remain optimistic that the identification of KIF18B as a potential prognostic marker and therapeutic target will contribute to the advancement of GBM treatment and improve patient outcomes.

## Data Availability

The original contributions presented in the study are included in the article/Supplementary Material, further inquiries can be directed to the corresponding author.
