# Supplementary material for: Study on the mechanism of KIF18B affecting the malignant progression of glioblastoma cells
Source: Front Genet. 2025 Mar 5;16:1540342. doi: 10.3389/fgene.2025.1540342 (PMC11919893; doi:10.3389/fgene.2025.1540342)
Supplement: Supplementary file 1 [file Table2.DOC]

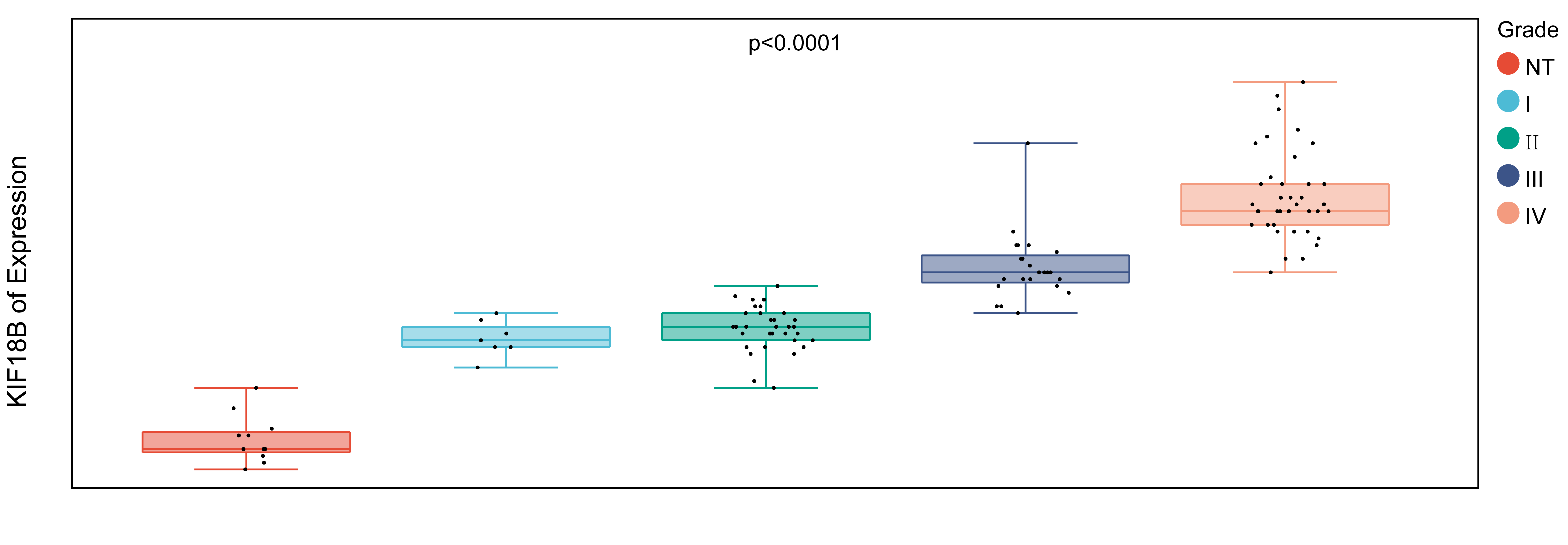
FIGURE S1

Expression of KIF18B in glioma of different grades. (A black dot represents a glioma tissue in the tissue chip).


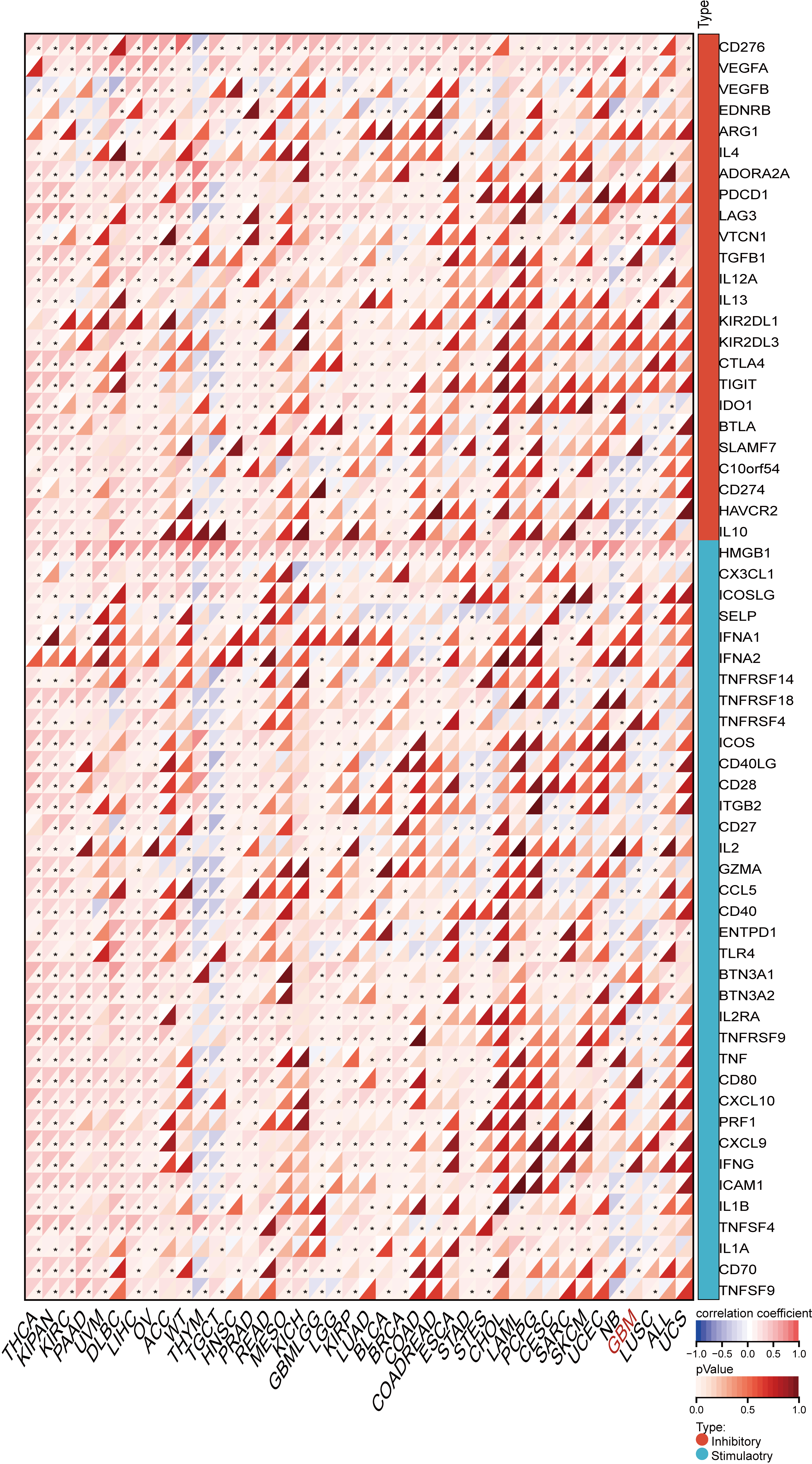
FIGURE S2

Correlation between KIF18B and 60 immune checkpoints (Inhibitory (24) and Stimulatory (36) marker genes). *p < 0.05; Red represents positive correlation, blue represents negative correlation.


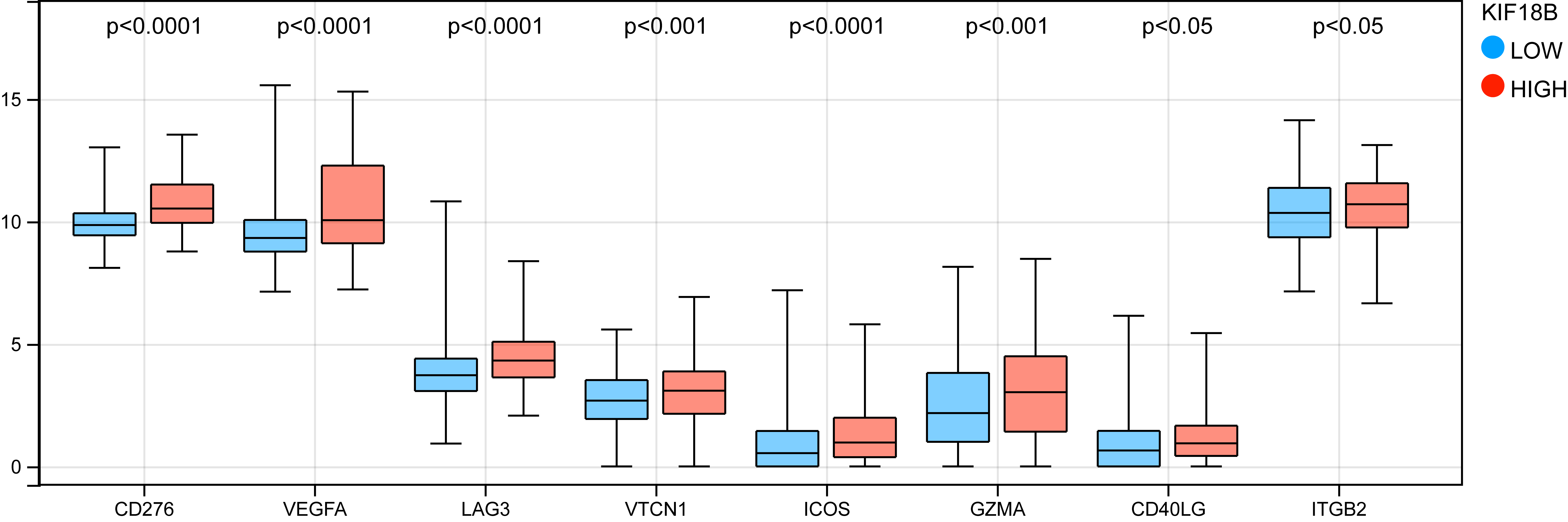


Figure S3

Differential expression of immune checkpoint molecules in groups with high or low KIF18B expression.


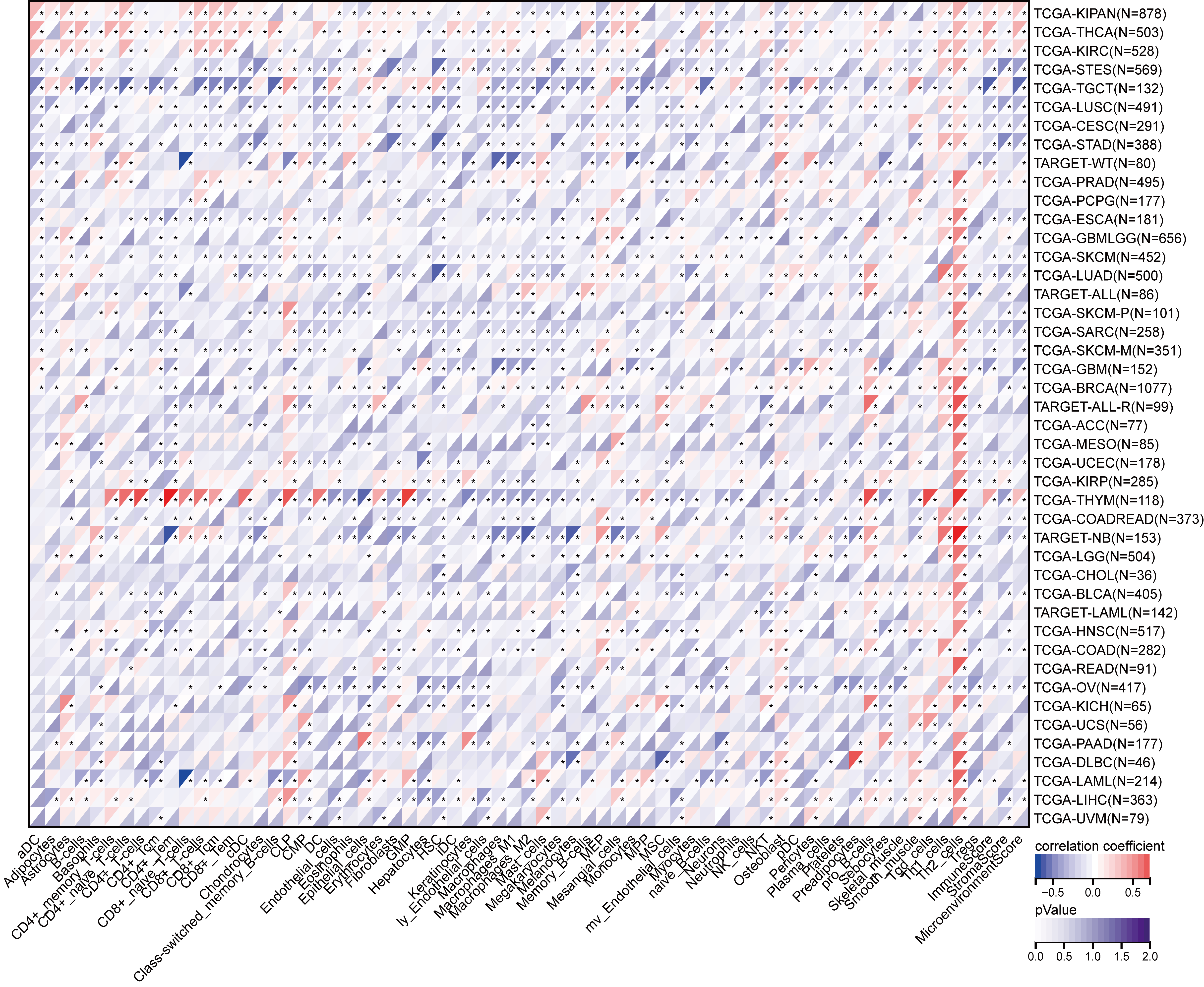


FIGURE S4

Correlation analysis of KIF18B mRNA expression with immune infiltration with xCELL algorithm. *p < 0.05; Red represents positive correlation, blue represents negative correlation.


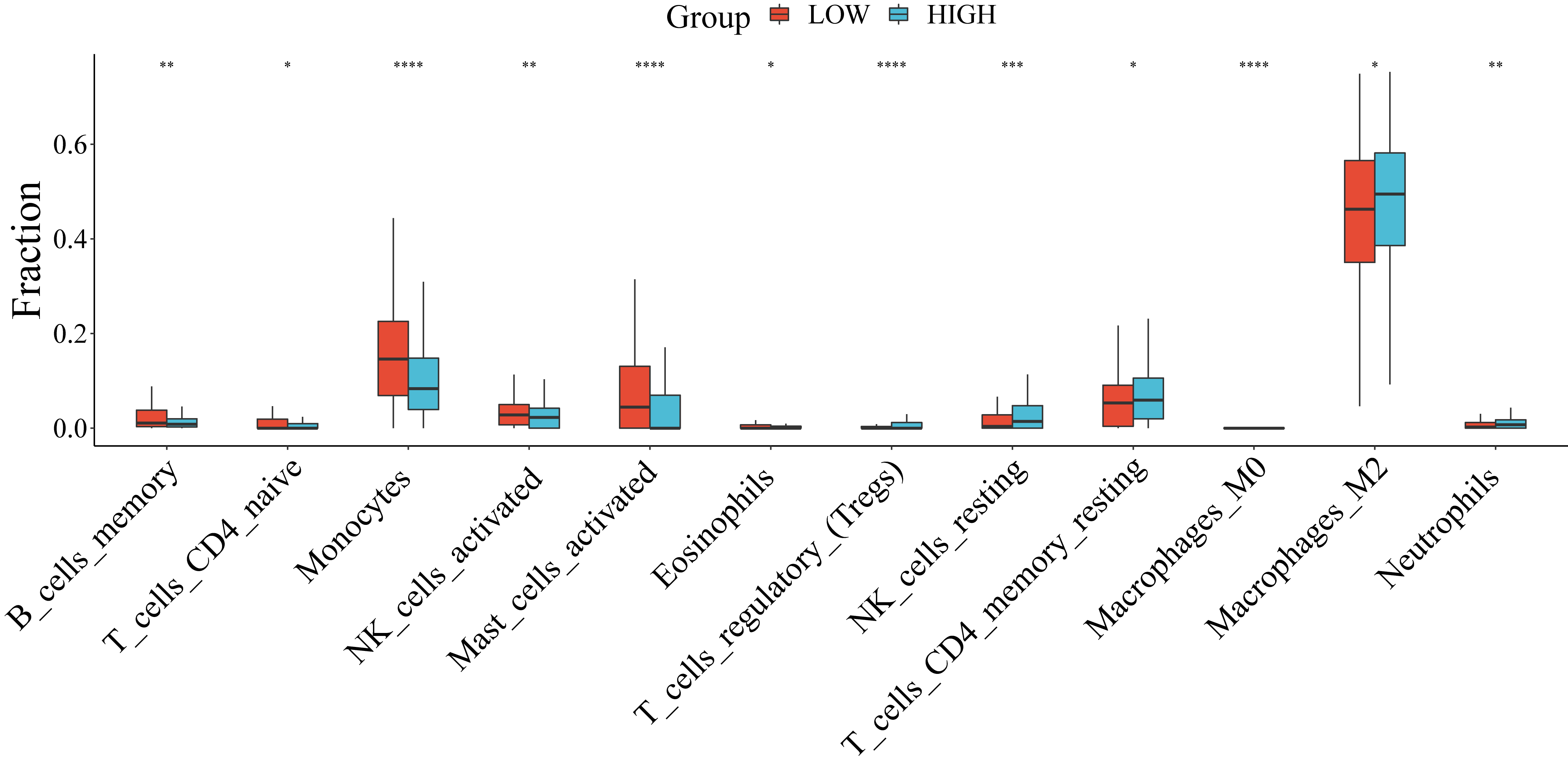


FIGURE S5

Correlation between the KIF18B expression level and infiltration of the tumor microenvironment with CIBERSORT algorithm. *p < 0.05, **p < 0.01, ***p < 0.001, ****p < 0.0001.
